# Clinical features and treatment outcome of non-small cell lung cancer (NSCLC) patients with uncommon or complex epidermal growth factor receptor (EGFR) mutations

**DOI:** 10.18632/oncotarget.15945

**Published:** 2017-03-06

**Authors:** Stefano Frega, Martina Lorenzi, Matteo Fassan, Stefano Indraccolo, Fiorella Calabrese, Adolfo Favaretto, Laura Bonanno, Valentina Polo, Giulia Zago, Francesca Lunardi, Ilaria Attili, Alberto Pavan, Massimo Rugge, Valentina Guarneri, PierFranco Conte, Giulia Pasello

**Affiliations:** ^1^ Department of Surgical, Oncological and Gastroenterological Sciences, University of Padova, Padova, Italy; ^2^ Department of Medicine, Surgical Pathology Unit, University of Padova, Padova, Italy; ^3^ Immunology and Molecular Oncology Unit, Istituto Oncologico Veneto IRCCS, Padova, Italy; ^4^ Department of Cardio-Thoracic and Vascular Sciences, University of Padova, Padova, Italy; ^5^ Medical Oncology, Azienda ULSS 9, Treviso, Italy; ^6^ Medical Oncology 2, Istituto Oncologico Veneto, IRCCS, Padova, Italy

**Keywords:** lung cancer, non-small cell, epidermal growth factor receptor, mutations, tyrosine kinase inhibitors

## Abstract

**Introduction:**

Tyrosine-kinase inhibitors (TKIs) represent the best treatment for advanced non-small cell lung cancer (NSCLC) with common exon 19 deletion or exon 21 epidermal growth factor receptor mutation (EGFRm). This is an observational study investigating epidemiology, clinical features and treatment outcome of NSCLC cases harbouring rare/complex EGFRm.

**Results:**

Among 764 non-squamous NSCLC cases with known EGFRm status, 26(3.4%) harboured rare/complex EGFRm. Patients receiving first-line TKIs (*N* = 17) achieved median Progression Free Survival (PFS) and Overall Survival (OS) of 53 (IC 95%, 2–105) and 84 (CI 95%, 27–141) weeks respectively, without significant covariate impact. Response Rate and Disease Control Rate (DCR) were 47% and 65%, respectively. Uncommon exon 19 mutations achieved longer OS and PFS and higher DCR compared with exon 18 and 20 mutations. No additional gene mutation was discovered by MassARRAY analysis. TKIs were globally well tolerated.

**Materials and methods:**

A retrospective review of advanced non-squamous NSCLC harbouring rare/complex EGFRm referred to our Center between 2010 and 2015 was performed. Additional molecular pathways disregulation was explored in selected cases, through MassARRAY analysis.

**Conclusions:**

Peculiar clinical features and lower TKIs sensitivity of uncommon/complex compared with common EGFRm were shown. Exon 19 EGFRm achieved the best TKIs treatment outcome, while the optimal treatment of exon 18 and 20 mutations should be further clarified.

## INTRODUCTION

The clinical knowledge of Epidermal Growth Factor Receptor (EGFR) molecular status and its therapeutic application, emerging in the early 2000s [1], has revolutionized non-small cell lung cancer (NSCLC) management, paving the way for targeted therapies. EGFR mutations (EGFRm) are typical of about 15% of NSCLC, mostly with adenocarcinoma histology, and quite peculiar of no smoker or former smoker patients [2].

Most EGFRm are strong predictors of response to tyrosine-kinase inhibitors (TKIs), with particular reference to the commonest E746-A750 deletion on exon 19 (ELREA) and L858R mutation in exon 21 [3].

Randomized phase III trials showed progression free survival (PFS) and response rate (RR) benefit of EGFR TKIs compared to platinum-doublets chemotherapy as first line treatment of EGFR mutated NSCLC [4–7], with subsequent post-hoc or preplanned subgroup analyses revealing higher benefit in cases harbouring classical exon 19 deletion [5–10].

Uncommon or rare EGFRm are all exons 18-21 alterations with the exception of common sensitizing cited above, or all those with a prevalence lower than 5% [11]. These gene alterations are in continuous discovery with variable frequency and sensitivity to targeted therapy [12].

Complex mutations are characterized by two or more different EGFRm in the same tumor sample, with heterogeneous prevalence reported [13–17]

The hypothetical carcinogenesis pattern of this special subset of EGFR-driven lung cancer, based on few *in vivo* and *in vitro* data, can be summarized in the principle “the unit is strength”: when a mutation with a low-intermediate oncogenic potential is not able itself to give rise to cancer, an additional one is required [13].

So far, no clinical trial has been designed to identify the best treatment for patients with these EGFR molecular variants, and maybe their relatively low incidence could constitute an obstacle to conceive it. Only post-hoc analyses of clinical trials, retrospective studies, case reports and *in vitro* findings addressed this issue, suggesting that uncommon EGFRm respond less to TKIs [12].

In a subgroup analysis of the NEJ002 study, the small number of patients with G719X or L861Q mutations (*N* = 5) treated with gefitinib showed shorter OS compared to those with common mutations[18]; data from a nationwide survey showed some activity of first generation TKIs gefitinib and erlotinib in patients with G719X-L861Q-S768I mutations, however with lower RR, PFS and overall survival (OS) compared with classical EGFR mutations [19].

A post-hoc analysis of combined LUX-Lung trials showed that afatinib achieves a median PFS of 10.7 months in patients with at least one of the three above mentioned uncommon mutations [20], not much lower than PFS seen in common mutations. We should consider that this patients group (*N* = 38) was heterogeneous, with almost half of them carrying a double mutation. An indirect proof of the apparent greater power of second generation TKIs in some uncommon EGFRm is their lower growth inhibitory concentration for *in vitro* growth arrest of cells transfected with exon 18 EGFR gene mutations [21].

If mutations discussed so far can be catalogued as partially sensitive, exon 20 mutations cluster encloses different types of modifications, [22, 23] including T790M with recognized resistance and low RR to first and second TKIs generation.

A wide subset of exon 20 insertions, whose mechanism of primary resistance are poorly understood, accounts as the third commonest de novo EGFR mutation: no shared consensus exists for treatment of such cases, for which chemotherapy regimens could represent a valid option. On the other hand, specifically designed third generation TKIs have been already proved to work against de novo or acquired T790M mutation, where resistance is principally due to increase in receptor affinity for adenosine triphoshate (ATP) [24]

Clinical reports about all other single point-mutations, in-frame deletions, in-frame duplications or insertions are anecdotal, as are those about patients with NSCLC harbouring complex EGFRm, representing the 6% of all EGFR mutated lung adenocarcinoma in a described Asian population [14].

The limited available data don't allow us to draw any conclusions about the optimal treatment of this special class of patients.

The aim of our observational retrospective study was to describe epidemiology, clinical features and treatment outcome of uncommon and/or complex EGFRm in a case series of NSCLC patients referring to our Institute. We also explored the coexistence of additional mutations in different genes in a patient subgroup having rare/complex EGFRm.

## RESULTS

### Epidemiology and clinical features of rare and complex mutations

Between 2010 and 2015, 992 patients with non-squamous NSCLC diagnosis referred to our Institute. EGFR mutational status was assessed in 764 patients; among these, no gene alteration was found in 675 (88%) cases, while 89(12%) subjects resulted EGFR mutated; assessment of mutational status was not performed in 228 cases which were excluded from the study.

**Figure 1:**
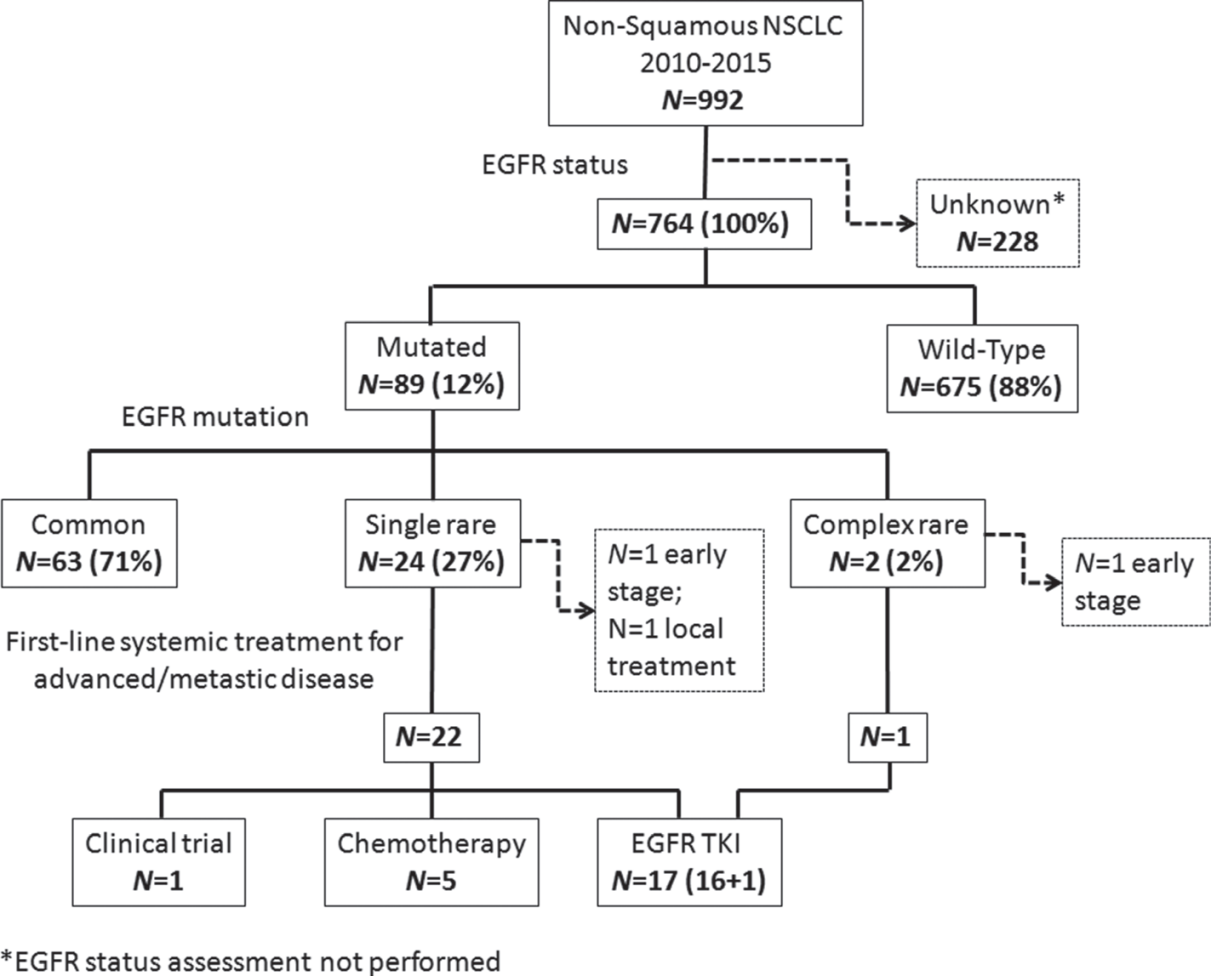
Consort diagram of the study population NSCLC: Non-Small Cell Lung Cancer; EGFR: Epidermal Growth Factor Receptor; TKI: Tyrosine Kinase Inhibitors.

Uncommon and complex EGFRm accounted for 3.4% of all non-squamous NSCLC cases. Among patients with EGFRm (*N* = 89), 63(71%) presented a common mutation (ELREA or L858R), 24 (27%) a rare isolated mutation and 2(2%) a complex mutation made of at least two rare mutations (Figure 1). Analyzing clinical features, median age was 68 year old (range, 47–86), with slight majority of females (*N* = 14, 54%). Sixteen (62%) patients received adenocarcinoma diagnosis, with histological subtypes mucinous/enteric in 6 (23%), acinar in 4 (15%), papillary in 2 (8%), poorly differentiated in 3 (12%), and lepidic in 1 (4%) cases; not otherwise specified (NOS) non squamous lung cancer was diagnosed in 10 (38%) cases. Smoking history was negative in half of the sample, while 12 patients (46%) have been exposed to smoke, equally divided in still smoker and former-smoker at diagnosis time; in an isolated case (4%) smoking status was unknown. At the diagnosis time, almost all patients presented in an optimal or good Eastern Cooperative Oncology Group (ECOG) performance status (PS), with 6 (23%) and 19 (73%) of them having 0 and 1 score respectively, despite the majority of them (*N* = 17, 65%) having stage IV. Advanced/metastatic stage was present in 24 patients at the diagnosis (*N* = 18, 70%) or subsequently to disease relapse (*N* = 6, 23%); metastatic spread were mostly in one (*N* = 10, 38 %) or two (*N* = 9, 35%) sites.

Most patients (*N* = 15, 58%) received only one systemic treatment line, while 8 (31%) patients received two or more treatment lines. Among the three patients (12%) not receiving any systemic treatment, two had an early stage disease and one had a relapse site suitable of loco-regional treatment (Table 1). No case of combined common mutations was observed. Among uncommon mutations, we observed a numerically homogeneous distribution in exon 18, 19 and 20 while no case of rare isolated mutation in exon 21 was found (Table 2).

**Table 1:** Main clinical and pathologic characteristics of 24 patients with rare/complex EGFR m

Clinical feature	*N* = 26 (100%)
*Gender*	
Male	12 (46%)
Female	14 (54%)
*Age*	
Median (range)	68 (47–86)
*ECOG score*	
0	6 (23%)
1	19 (73%)
2	0 (0%)
3	1 (4%)
*Smoking status*	
Current Smoker	6 (23%)
Former smoker	6 (23%)
Non smoker	13 (50%)
Unknown	1 (4%)
*Histology*	
Lepidic	1 (4%)
Acinar	4 (15%)
Papillary	2 (8%)
Mucinous	4 (15%)
Enteric	2 (8%)
Poorly differentiated	3 (12%)
Not otherwise specified (NOS)	10 (38%)
*Stage at diagnosis*	
Stage I	4 (15%)
Stage II	3 (12%)
Stage III	2 (8%)
Stage IV	17 (65%)
*Surgery*	
Radical	7 (27%)
Radical and palliative	1 (4%)
Palliative	
Palliative	2 (8%)
Diagnostic	2 (8%)
None	14 (54%)
*Neoadjuvant/adjuvant therapy*	
Yes	5 (19%)
No	21 (81%)
*Advanced disease*	
Yes, at diagnosis	18 (69%)
Yes, at recurrence	6 (23%)
No	2 (8%)
*Metastatic sites*	
0	3 (12%)
1	10 (38%)
2	9 (35%)
3	3 (12%)
4	1 (4%)
*Treatment lines*	
One	15 (58%)
Two	6 (23%)
Three	2 (8%)
None	3 (12%)
*Radiotherapy*	
Palliative	4 (15%)
Adjuvant	1 (4%)
None	21 (81%)

**Table 2:** Rare and complex genetic alterations in EGFR gene kinase domain

Exon	Mutation type	Aminoacid change	N	% of total mutated
*Exon 18*	Point mutations	E709K/Q/Stop	3	3%
		G719A/C/S	4	4%
*Exon 19*	Insertions	I745insKIPVAI	1	1%
		L747-S752; insP	1	1%
	Insertions/ Deletions	del747-K754insSR	1	1%
		del L747-P753insQ	1	1%
		delE746-S752insV	1	1%
		delL747-P753insS	2	2%
	Deletions	L747-K754	1	1%
		S752-I759	1	1%
*Exon 20*	Insertions	H773 ins HPH	1	1%
		V769insASV	1	1%
		D770insSVD	1	1%
	Duplications	Asn771_His773dup	1	1%
	Point mutations	S768R	1	1%
		H773G	1	1%
		Q812R	1	1%
	Not specified	-	1	1%
*Exons 18 plus 21*	Combined point mutations	E709K L833V H835L	1	1%
*Exons 18 plus 20*	Combined point mutations	G719A V769M	1	1%

### Treatment outcome

All patients with advanced/metastatic disease (*N* = 23, 95%) received a systemic treatment. First or second generation TKIs were prescribed in 17 (71%) patients: gefitinib in 11 (46%), erlotinib in 3 (12.5%) and afatinib in 3 (12.5%) cases. A platinum-based chemotherapy with or without an anti-angiogenic drug was administered to 5 (21%) patients; one (4%) patient was treated with an anti-programmed cell death protein 1 (PD-1) monoclonal antibody in the context of a randomized clinical trial (Figure 1).

In the group of patients receiving a systemic treatment for advanced/metastatic disease, we observed 8(35%) cases of partial response(PR) and 8 (35%) cases of stable disease(SD), 7 (30%) cases of progressive disease(PD) and no case of complete response(CR). RR was 35% (95% confidence interval CI; 16%–57%), while disease control rate(DCR) was 70% (95% CI; 47%–87%). All patients harboring a resistance exon 20 EGFR mutation, who received first-line chemotherapy, achieved a stable disease.

Among patients who received an EGFR TKI (*N* = 17) we observed 8 (47%) PR, 3 (18%) SD and 6 (35%) PD; RR was 47% (95% CI; 23%–72%) with DCR of 65% (95% CI; 38%–86%) (Table 3).

**Table 3:** Clinical features and best radiological response to treatment of each patient harbouring rare and/or complex mutations

Sex	Age (years)	Smoking status	Histology	Mutation	1°line therapy	Best response
M	71	Former	NOS	ex 18 E709K ex 21 L833V/H835L	afatinib	PR
F	69	Current	Acinar	ex 18 G719A ex 20 V769M	-	-
M	56	No	Mucinous	ex 19 I745insKIPVAI	erlotinib	SD
F	68	Former	NOS	ex 20 Q812R	CT	PD
F	68	Former	Acinar	ex 20 D770insSVD	-	-
M	62	No	NOS	ex 20 (His773G)	CT	SD
M	54	Former	Papillary	ex 20 (unspecified)	CT	SD
F	56	No	Mucinous	ex 20 Asn771_His773dup	erlotinib	PD
F	66	Current	Poorly diff.	ex 20 H773 ins HPH	erlotinib	PD
M	73	Current	Poorly diff.	ex 18 E709K	gefitinib	PD
M	86	No	NOS	ex 18 E709Stop	gefitinib	PD
F	71	Unknown	Mucinous	ex 19 del747-K754insSR	gefitinib	SD
F	79	No	Mucinous	ex 20 S768R	gefitinib	PD
F	56	Current	Mucinous	ex 19 del L747-P753insQ	gefitinib	SD
M	68	No	Lepidic	ex 18 E709Q	-	-
M	51	No	Acinar	ex 19 delE746-S752insV	gefitinib	PR
F	74	No	Poorly diff.	ex 19 delL747-K754	afatinib	PR
M	53	No	Mucinous	ex 18 G719A	CT	SD
F	64	Unknown	NOS	ex 20 V769insASV	CT	SD
F	76	No	Papillary	ex 19 del S752-I759	gefitinib	PR
M	60	Former	Acinar	ex 18 G719C	gefitinib	PR
F	73	No	NOS	ex 19 L747-S752;insP	gefitinib	PR
M	79	Former	NOS	ex 18 2155G>A, pGly719Ser	gefitinib	PD
F	75	No	NOS	ex 19 delL747-P753insS	gefitinib	PR
M	54	Current	NOS	ex 19 delL747-P753insS	afatinib	PR
F	47	Current	NOS	ex 18 G719A	CT	SD

M: Male; F: Female; Diff: differentiated; NOS: not otherwise specified; ex: exon; CT: chemotherapy; PR: partial response; SD: stable disease; PD: progressive disease

**Figure 2:**
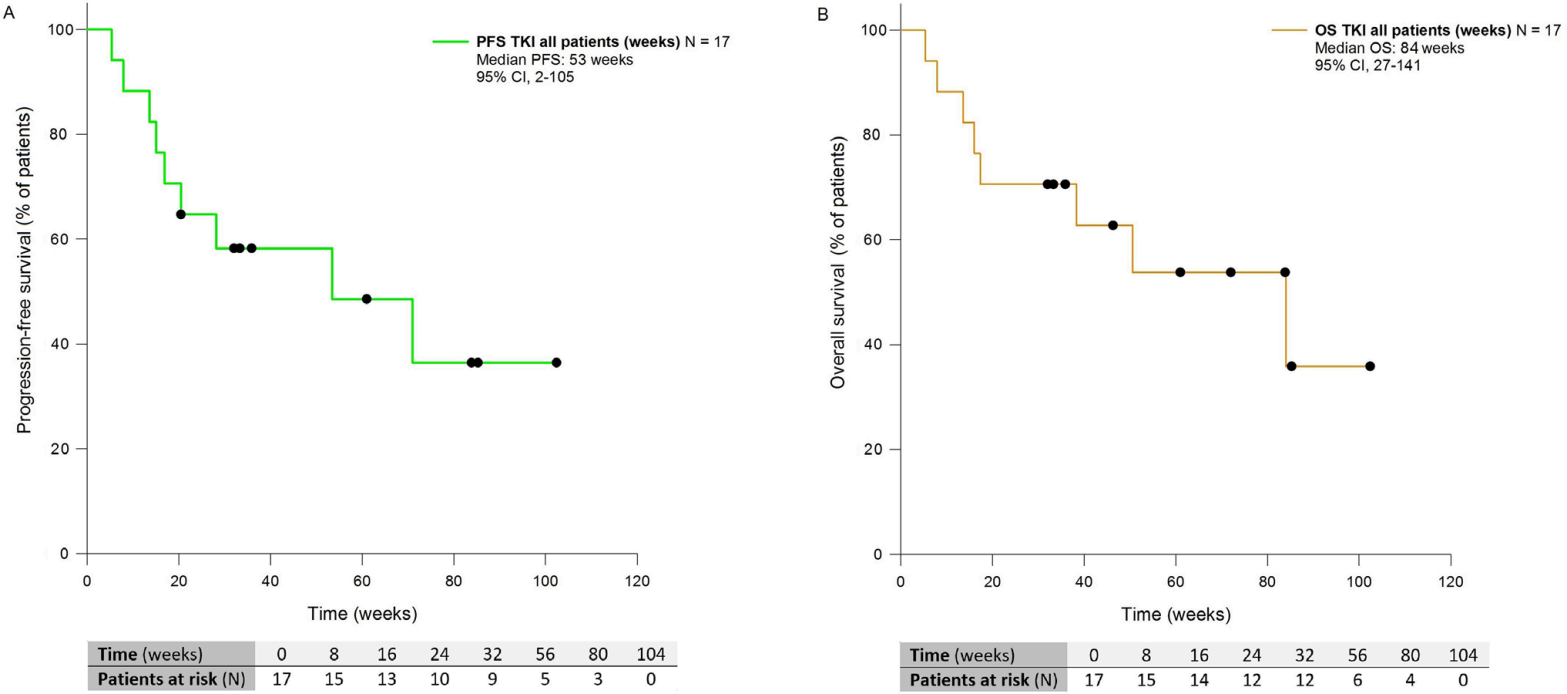
Kaplan-Meier curves showing PFS (**A**) and OS (**B**) of patients with NSCLC harboring rare and complex EGFR mutations and receiving EGFR TKIs front-line. PFS: Progression-free Survival; OS: Overall Survival; TKI: Tyrosine kinase Inhibitors; CI: Confidence Interval.

With a median follow-up of 38 weeks, median PFS and OS in patients treated with first-line EGFR TKIs were 53 weeks (CI 95%, 2–105) and 84 weeks (CI 95%, 27–141) respectively (Figure 2).

Longer PFS and OS were shown in patients treated with first-line EGFR TKIs compared with chemotherapy, even though without statistical significance, likely because of the small sample size (Supplementary Figure 1).

We observed an isolated PR (25%) in the subgroup 1, and 3 (75%) cases of PD at the first radiological assessment; 6 (67%) patients reported PR and 3 (33%) a SD in the subgroup 2; all three patients within the subgroup 3 had PD. No significant differences in terms of RR were observed between subgroups, while higher DCR was shown in subgroup 2 compared with subgroup 1 (*p* = 0.014) and 3 (*p* = 0.0045) (See Supplementary Figure 2). The only patient harbouring a complex mutation 3 (subgroup 4) showed a partial response to TKIs.

**Figure 3:**
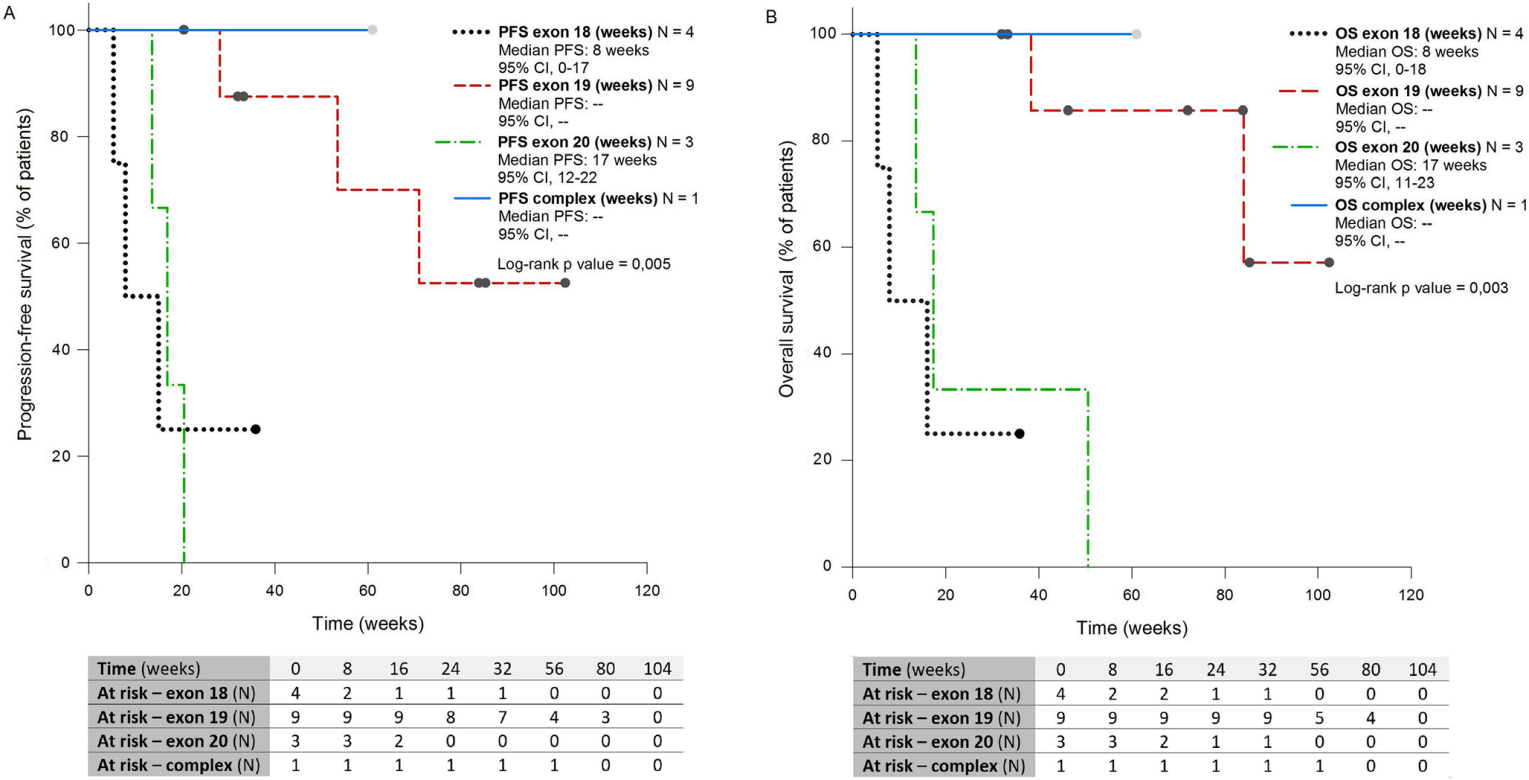
Kaplan-Meier curves showing PFS (**A**) and OS **(B**) of patients with NSCLC harboring rare and complex EGFR mutations, receiving EGFR TKIs front-line, of four subgrups defined according to mutation type. PFS: Progression-free Survival; OS: Overall Survival; CI: Confidence Interval

PFS was significantly longer in subgroup 2 (median PFS: not reached) compared with subgroup 1 (median PFS 8 weeks, *p* = 0.037, CI 95%) and subgroup 3 (median PFS 17 weeks, *p* = 0.0009, IC 95%) (Figure 3A).

Rare mutations on exon 19 were also predictive of longer survival (median OS: not reached) compared with rare mutations in exon 18 (mwedian OS 8 weeks, *p* = 0.014, CI 95%) or exon 20 (median OS 17 weeks, *p* = 0.014, CI 95%) (Figure 3B)

Median PFS and OS were not reached in the subgroup 4.

No covariate significantly impacts on PFS and OS at the univariate (See Supplementary Table 1) and multivariate (See Supplementary Table 1) analyses, even though data should be interpreted with caution, due to the limited sample size.

### Treatment tolerability

An adverse event (AE) of any grade was observed in 13 (76%) patients. Maximum grade (G) of AE was 1 in 5 (33%) patients, G2 in 6 (35%) patients, and G3 in 1(6%) patient receiving gefitinib.

Temporary treatment interruption for any cause was registered in 5 (29%) patients, while interruptions due to drug-related AE were 2 (11%), both in patients receiving afatinib. No case of permanent treatment discontinuation was observed.

Dose reduction was required only in one patient (5%) taking afatinib. More frequently observed AE were diarrhea (*N* = 8, 47%), paronychia (*N* = 7, 41 %), asthenia (*N* = 4, 24 %), mucositis (*N* = 2, 12 %) with isolated cases of skin dryness, corneal erosion, urinary tract infection and nausea (See Supplementary Table 3).

The incidence of any drug-related AE was higher for afatinib (*N* = 3, 100%), than for gefitinib or erlotinib (*N* = 8, 73% and *N* = 2, 67% respectively). No statistically significant difference in terms of AE between patients receiving erlotinib compared to those treated with two other TKIs (*p* = 1.00), and between first and second generation TKIs (*p* = 1.00) were observed.

### Exploratory analysis of additional molecular alterations in genes other than EGFR

We searched for additional molecular alterations in other genes as possible driver of tumor progression, in the subgroup of cases (*N* = 6) with complex EGFRm or with rare EGFR mutations not responsive to first-line EGFR TKIs. Analyzed genes by multiplex PCR sequencing (Sequenom) included kras, braf, nras, pi3kca, alk, erbb2, ddr2, mapk1 and ret. MassARRAY analysis confirmed EGFR mutations but did not disclose additional mutations.

## DISCUSSION

Looking at results of available phase III clinical trials, EGFRm in NSCLC represent a strong predictive factor to EGFR TKIs. However, most studies consider only more frequent EGFRm, namely the deletion (ELREA) in exon 19 and the point mutation L858R in exon 21. On the contrary, there are no clear data about epidemiology, clinical features and drugs efficacy in patients harboring uncommon mutations. Survival results in patients with uncommon gene alterations, extrapolated from few randomized clinical trials comparing targeted therapies *versus* chemotherapy (IPASS, NEJGSG002, LUX-LUNG 3 e LUX-LUNG 6), [8, 18, 20] provide some information but the small sample size and the heterogeneous clinical impact of such mutations limit their application in the ‘real-life’ practice. These critical issues were the basis for the design of this retrospective study on a series of patients referred to our Center. The descriptive purpose of the study offers some useful evidence about patient management based on clinical practice, especially considering how difficult could be to realize prospective studies restricted to subjects with rare and/or complex mutations. In our study, 12% of lung adenocarcinomas carried an EGFR mutation, a prevalence in line with literature data in Caucasian populations.[2, 28].

Uncommon and complex mutations accounted for less than 4% of all non-squamous NSCLC cases. Among cases carrying EGFRm, 71% had a common mutation and 27% a rare mutation, while 2% a complex mutation. The percentage of rare mutations is slightly higher than in other studies; [15, 18] similar frequencies were only identified in an Australian study, in which the incidence was about 25% of a small sample size. [29] Instead it's not yet completely elucidated the incidence of complex mutations, ranging from 3 to 25%, [13, 15–17] as well as their most plausible role in malignancies development: the appealing sequential selection of suboptimal mutation model, for example, doesn't clarify the meaning of an additional mutation combined to a common one [30].

We reported a prevalence of G719X and E709X point mutations in exon 18 (7%) and insertions/duplications in exon 20 (4%) in line with previous studies [21, 28, 31, 32] while the prevalence of exon 19 insertions (5%) were quite higher than that reported in the literature data [33]. No rare mutation in exon 21 alone was observed; on the contrary two synchronous exon 21 point mutations (L883V/H835L) have been found in a subject with triple complex mutation [34], a previously detected finding by other groups [35].

With regard to the clinical characteristics of studied subjects, we observed a considerable incidence of acinar and mucinous/enteric subtypes (15% and 23% respectively), in contrast with literature data reporting no EGFRm in mucinous subtype.[36, 37] This is an interesting finding considering the increasing incidence of such histologic subtype, probably because of the implementation of the lung adenocarcinoma diagnostic and classification process, and the lack of data on the optimal treatment of this kind of aggressive neoplasia poorly responsive to standard chemotherapy. The percentage of patients currently exposed to cigarette smoke in our series (23%) is superior than reported for common sensitizing mutation (about 6%), while the percentage of current and previous smokers (46%) is only slightly higher. [38] This finding was reported in two other European studies: Lohinai et al. showed a statistically significant association between rare EGFRm and smoking habits [39], while Pallis et al. showed that the percentage of smokers is higher, although not significantly, in uncommon EGFRm than in classical one. [40] Furthermore, Beau-Faller et al. reported a higher frequency of exon 18 mutations in smokers [28]; in our study, patients with exon 18 mutations (rare or complex) showed smoking exposure in six of nine cases (67%). The response rate (RR) of patients with rare and complex mutations treated with TKIs was 47% (95% CI; 23%–72%), while the rate of disease control (DCR) was 65% (95 % CI; 38%–86%). Similar findings have already been reported in other studies [41] in which RR to TKIs varies between 34% and 50% and the DCR between 57.4% and 75.6%, in any case lower than those of common mutations for which RR is greater than 60% for both first and second generation drugs.

However, with the obvious limits of an indirect comparison, survival parameters of our patients with rare EGFRm seemed more encouraging than those of patients with similar molecular characteristics, previously published [42]. In particular, median PFS and OS in TKI treated sample were of 13 and 20 months, respectively. These values didn't reach anyway outcome data reported in the first-line pivotal trials [5, 8] comparing TKIs *versus* chemotherapy in patients harboring common EGFR alterations. Moreover, real-life data from a subset of mutated patients referred to our Institute showed a median PFS in uncommon mutations group not significantly lower than in common mutation group, where median PFS was 14 months (15 months and 9 months in patients harboring exon 19 deletions and exon 21 mutations, respectively) (Supplementary Figure 3). On the same way, DCR in our case series harbouring common mutations was 90% (57% PR and 33% of SD), not significantly higher than DCR in subgroup 2 (data not shown).

The heterogeneous prevalence of different uncommon mutations might explain such controversial results, as much as the retrospective nature of most case series.

We also analyzed the predictive role of different uncommon mutations by dividing patients into four subgroups. We observed higher benefit in terms of DCR, median PFS and OS in the subgroup carrying exon 19, compared with exon 18 and 20 mutations.

Few data on the exon 19 insertions seem to confirm the positive predictive role of these mutations [11]: as reported by He et al. mutated cells are sensitive to TKIs both *in vitro* and *in vivo*, thus constituting a new family of sensitizing mutations. [33] Patients harboring exon 18 mutations (E709X and G719X) achieved shorter median PFS and OS with only one case of PR to gefitinib. These findings agree with published results, reporting low, although heterogeneous, response rates (0% to 37%) and dismal prognosis (median PFS from 1.3 to 6.3 months).[19, 28, 41] A post hoc analysis of NEJ002 study showed lower efficacy of gefitinib than chemotherapy in people with G719X and L861Q mutations, without significant differences between the two mutations [18]. In contrast, in subjects with G719X mutation (alone or in combination) treated with afatinib both RR and median PFS (77.8% and 13.8 months, respectively) are greater than those receiving chemotherapy [9], thus suggesting second-generation TKIs as a possible option in this mutations class [21]. Survival data of subgroup 3 were significantly lower compared to subgroup 2 but not subgroup 1, probably due to the limited sample size. Our study confirms the primary TKIs resistance in patients carrying exon 20 alterations, with all (*N* = 3) cases developing PD as best response. All literature data about exon 20 mutations show a very low RR (about 8%) and poor median PFS (about 2.5 months) with first two generation TKIs [9, 41]. Few data instead exist on isolated point mutations, in particular the S768X, which might confer an intermediate sensitivity to targeted therapy [19]; in our study, however, only one patient had the above mutation (S768R) and did not achieve any response to therapy.

Unlike the other subgroups, the most appropriate treatment in these patients might be chemotherapy, as confirmed by disease stabilization achieved in our case series. The only patient with complex mutation treated with an irreversible TKI showed a good response and survival. Data about this particular kind of subpopulation are lacking, with an isolated study showing poor RR, however without translating into a survival reduction.[43]

Molecular cancer heterogeneity is decisive, since treatment effectiveness appears to depend on the specific mix of carried mutations: RR and PFS seem better [44] in presence of a common mutation, and increase further, in case of co-presence of the two commonest mutations. Targeted drugs appear to be the best therapeutic option for these patients.

Despite an overall incidence of any grade AE of 78% with TKIs, a single serious AE (G3 diarrhea) was recorded. Even though no significantly different incidence of AE was shown among the three drugs, we confirmed their peculiar safety profile and showed more frequent dose reduction or temporary treatment interruption with afatinib;[45, 46] anyway there was no case of definitive therapy discontinuation for AEs. Overall, our data confirmed TKIs as a well tolerated treatment, with a peculiar toxicity profile manageable with supportive care or dose reductions.[47] MassARRAY analysis did not detect additional alterations in driver genes in this small subgroup of cases. It can be assumed that the EGFRm are important drivers for tumor growth and that, in subjects non-responsive to TKIs therapy, primary resistance is due to the specific EGFR alterations, rather than being accounted for by other concomitant mechanisms. These hypotheses should, however, be confirmed in larger series. The main limitations of this study consist in the small number of patients involved, due to the rarity of the condition, and the retrospective nature of the study.

Even though a prospective multicenter study should be performed in order to propose a treatment algorithm in such uncommon or complex EGFRm, clinical cases or series report acquire their own relevance in the context of clinical practice.

## MATERIALS AND METHODS

### Patient population

Data from patients with histological diagnosis of non-squamous lung cancer have been collected retrospectively from January 2010 to December 2015. Histological samples were obtained with surgical excision, surgical biopsy, trans-bronchial or computerized tomography (CT) scan guided biopsy of primary tumor, surgery or biopsy of metastatic sites. All subjects with at least one rare and/or complex EGFRm were included. A brain-thorax-abdomen CT scan was performed before starting systemic treatment for advanced/metastatic disease with successive radiological assessment every 2-3 months, according to clinical practice. Before study inclusion, all patients signed the informed consent form, according to privacy protective rules for recording clinical data for research and study purposes. The following clinical data were collected: gender, age, smoking status, histology, disease stage at diagnosis according to 7^th^ edition Classification of Malignant Tumors (TNM) of NSCLC [25], metastatic sites at diagnosis or at disease recurrence. A subject was classified as former-smoker if smoking habits interrupted by at least one year.

### Mutational analysis

Mutational analysis of exon 18–21 of EGFR gene was performed at the time of histological diagnosis and in any case before the start of first line systemic treatment, through Sanger sequencing, PCR-based methods, or pyrosequencing. In the subgroup of patients with complex or rare mutations not responsive to first or second generation TKIs, the mutational status of genes different from EGFR has been investigated by massARRAY (Sequenom) analysis; investigated genes included kras, braf, nras, pi3kca, alk, erbb2, ddr2, mapk1 and ret.

### Treatment

First line systemic treatment of patients with advanced/metastatic disease was administered according to national and international guidelines. In particular, a first (erlotinib or gefitinib) or second (afatinib) generation TKI was prescribed in cases with EGFRm with unknown predictive value. Patients harboring EGFRm more frequently associated with resistance to targeted therapy (for example, exon 20 insertions) have been treated with standard chemotherapy (platinum-based doublet *plus* pemetrexed or gemcitabine or paclitaxel; platinum-based triplet *plus* gemcitabine and bevacizumab) or were included in clinical trials, when available. TKIs were administered *per os* daily with initial dose of 150 mg, 250 mg and 40 mg for erlotinib, gefitinib and afatinib respectively, until disease progression or death, unacceptable toxicity or patient refusal. Drug choice was at the physician discretion.

### Efficacy and safety assessment

Best radiological response by CT imaging was assessed in all patients who received a systemic treatment for advanced/metastatic disease, and classified in CR, PR, SD and PD according to Response Evaluation Criteria In Solid Tumors (RECIST) 1.1 criteria [26]. Best response rate (RR) was calculated as the proportion of subjects with CR and PR to treatment, while disease control rate (DCR) was calculated as the proportion of patients with CR, PR and SD.

Progression free survival (PFS) to first line therapy was measured as the time between diagnosis of advanced disease and first evidence of progression during or after first line therapy, or death for any cause; overall survival (OS) was measured as the time elapsed from diagnosis of advanced/metastatic disease and death for any cause. Best radiological response and survival data were reported for the intention-to-treat population and for the subgroup of patients receiving a first line TKI.

We also assessed TKIs best response and survival in four patient subgroups, according to the mutation type: subgroup 1 (rare mutations in exon 18); subgroup 2 (rare mutations in exon 19); subgroup 3 (rare mutations in exon 20); subgroup 4 (complex mutations).

All AE were registered and graded through Common Terminology Criteria for Adverse Events (CTCAE) version 4.0. [27] in patients treated with first line TKIs.

### Statistical analysis

RR and DCR binomial proportion confidence interval was calculated with Clopper-Pearson exact test. Different response rates among patient subgroups were analyzed with Fisher exact test. PFS and OS curves were designed with Kaplan-Meier method. PFS was analyzed according to these covariates: age (< 70 *versus* ≥ 70 years old), stage (IIIb *versus* IV), ECOG PS (0 *versus* ≥ 1), best response to first line therapy (PR/SD *versus* PD), number of metastatic sites (< 3 *versus* ≥ 3), smoking status (exposed *versus* not exposed), TKI generation (first *versus* second), histological subtype (mucinous/enteric *versus* not mucinous/enteric). The same variables were considered in OS analysis, with the addition of data about any subsequent treatment lines (yes *versus* no). Log-rank test and Cox proportional risk model were used to estimate the impact of such variables on PFS and on OS. Differences of adverse events among different TKIs were analyzed with exact Fisher test. Statistical analyses were performed through SigmaPlot software.

## SUPPLEMENTARY MATERIALS FIGURES AND TABLES



## Abbreviations

AEs: Adverse Events
CR: Complete Response
CTCAE: Common Toxicity Criteria for Adverse Events
DCR: Disease Control Rate
ECOG: Eastern Cooperative Oncology Group
EGFR: Epidermal Growth Factor Receptor
GEFRm: EGFR mutations
ELREA: E746-A750 deletion on exon 19
G: Grade
NSCLC: Non-Small Cell Lung Cancer
OS: Overall Survival
PD: Progressive Disease
PFS: Progression Free Survival
PR: Partial Response
PS: Performance Status
RECIST: Response Evaluation Criteria In Solid Tumours
RR: Response Rate
SD: Stable Disease
TKIs: Tyrosine Kinase Inhibitors
