# Supplementary material for: Clinical features and treatment outcome of non-small cell lung cancer (NSCLC) patients with uncommon or complex epidermal growth factor receptor (EGFR) mutations
Source: Oncotarget. 2017 Mar 6;8(20):32626–38. doi: 10.18632/oncotarget.15945 (PMC5464815; doi:10.18632/oncotarget.15945)
Supplement: Supplementary file 1 [file oncotarget-08-32626-s001.pdf]

Clinical features and treatment outcome of non-small cell lung cancer (NSCLC) patients with uncommon or complex epidermal growth factor receptor (EGFR) mutations

Supplementary Materials

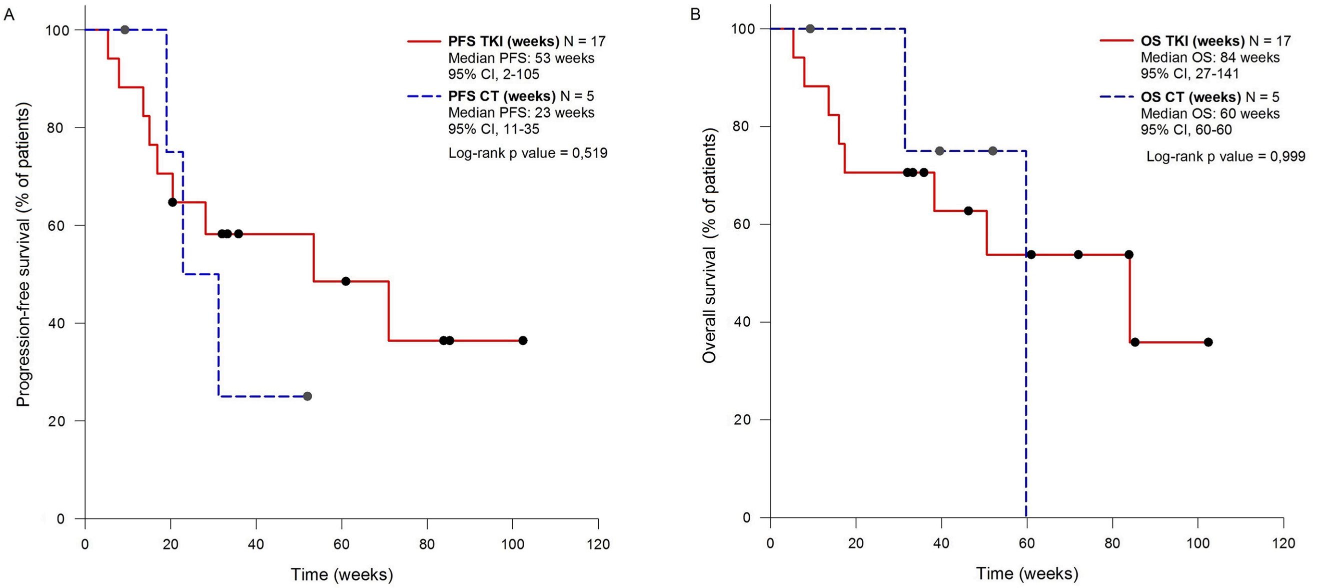

**Time** (weeks)	0	8	16	24	32	56	80	104
**Patients at risk – TKI** (N)	17	15	13	10	9	5	3	0
**Patients at risk – CT** (N)	5	5	4	2	1	0	0	0

**Time** (weeks)	0	8	16	24	32	56	80	104
**Patients at risk – TKI** (N)	17	15	14	12	12	6	4	0
**Patients at risk – CT** (N)	5	5	4	4	3	1	0	0

Supplementary Figure 1: Kaplan-Meier curves showing PFS (**A**) and OS (**B**) of patients with NSCLC harboring rare and complex EGFR mutations, receiving EGFR TKIs versus chemotherapy front-line. PFS; Progression-free Survival; OS: Overall Survival; TKI: Tyrosine kinase Inhibitor; CT: Chemotherapy; CI: Confidence Interval.

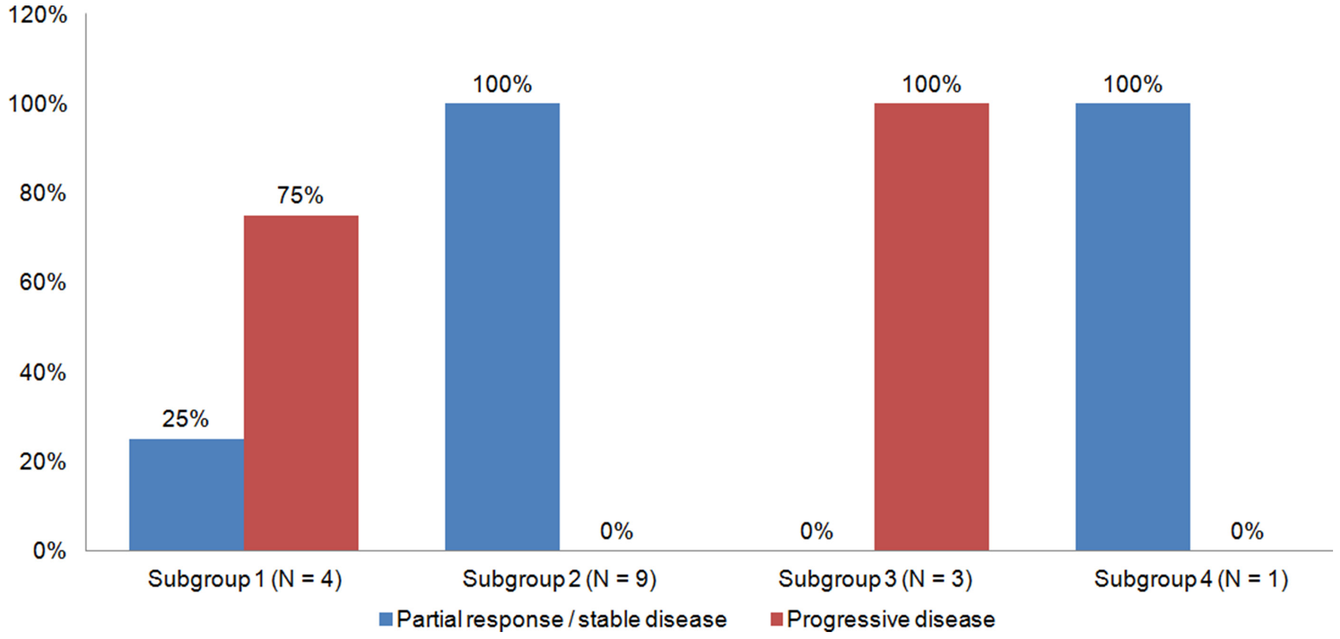

Supplementary Figure 2: Best response to TKI treatment in four subgroup of patients defined according to mutation type.

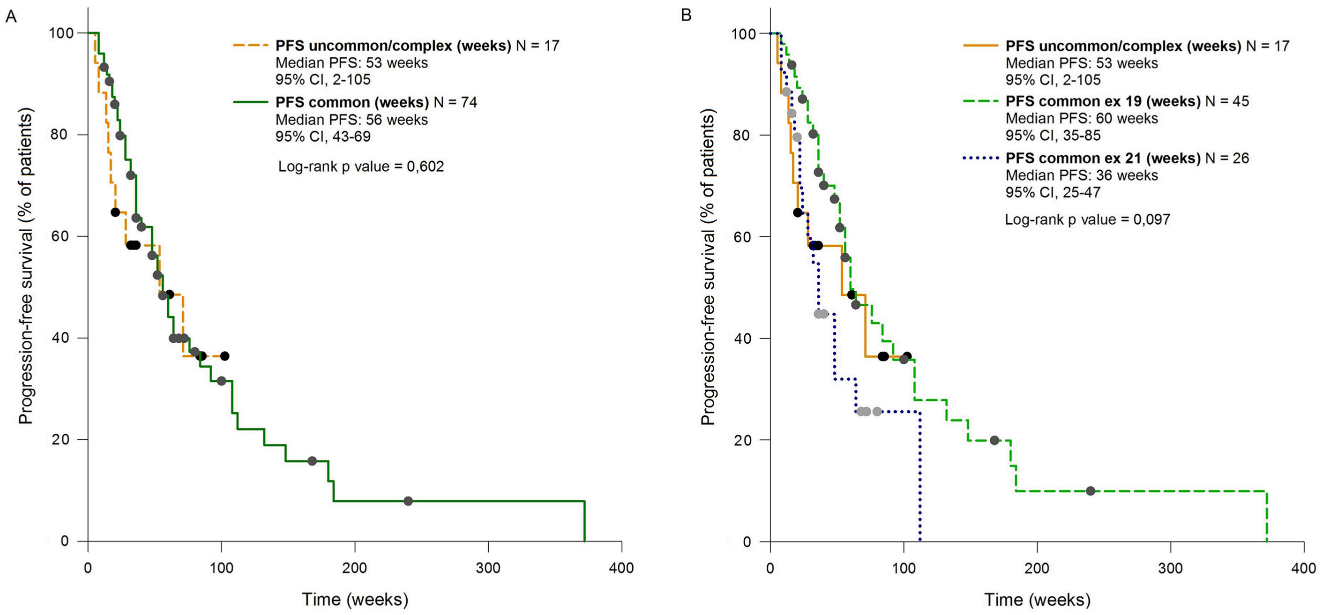

Time (weeks)	0	8	16	32	56	80	104	128	240	380
At risk – uncommon/complex (N)	17	15	14	9	5	3	0	0	0	0
At risk – common (N)	74	74	66	48	26	14	10	7	2	0

Time (weeks)	0	8	16	32	56	80	104	128	240	380
At risk – uncommon/complex (N)	17	15	14	9	5	3	0	0	0	0
At risk – common ex 19 (N)	48	48	45	36	21	12	1	0	0	0
At risk – common ex 21 (N)	26	26	21	12	5	2	9	7	2	0

Supplementary Figure 3: Kaplan-Meier curves showing PFS of patients with NSCLC harboring EGFR mutations and receiving EGFR TKIs front-line, according to mutational status. In particular, comparison between patients with common and uncommon/complex mutations (**A**) and between patients with common mutations in exon 19, exon 21 and uncommon/complex mutations (**B**). PFS: Progression-free Survival; CI: Confidence Interval.

Supplementary Table 1: Impact of covariates at the univariate analysis (logRank test) on PFS and OS in patients treated in first line with TKIs

Covariate	*N*	PFS (*p* value)	OS (*p* value)
Smoke exposure			
Yes	7	0.924	0.952
No	10		
Hystological subtypes			
Mucinous/Enteric	5	0.761	0.931
Not mucinous/Enteric	12		
TKI drug			
Erlotinib	3	0.671	0.767
Gefitinib	11		
Afatinib	3		
TKI drug generation			
First generation	14	0.928	0.571
Second generation	3		

NOS, Not otherwise specified; TKI: tyrosine kinase inhibitor; PFS: progression free survival; OS: overall survival.

Supplementary Table 2: Impact of covariates at the multivariate analysis (Cox model) on PFS and OS in patients treated in first line with TKIs

Covariate	PFS (*p* value)	OS (*p* value)
Stage<br>IIIb vs IV	1.000	NS
Number of metastatic sites<br>< 3 vs ≥ 3	0.979	NS
ECOG PS<br>0 vs ≥ 1	0.981	NS
Age (years old)<br>< 70 vs ≥ 70	0.986	NS
Smoke exposure<br>Yes vs No	0.977	NS
TKIs generation<br>First generation vs second generation	0.977	NS
Best response<br>PR/SD vs PD	0.976	NS
Hystological subtypes<br>Mucinous/enteric vs not mucinous/enteric	0.977	NS
Subsequent treatment lines after first-line<br>Yes vs No	NE	NS

TKIs, tyrosine kinase inhibitors; PR, Partial response; SD, Stable disease; PD, Progressive disease; PFS, Progression free survival; OS, Overall survival; NS, not significant; NE, not evaluable.

Supplementary Table 3: Adverse events of any grade registered in subjects treated with TKIs

Adverse event	*N* (%treated)	*N* (%) Gefitinib	*N* (%) Erlotinib	*N* (%) Afatinib
Diarrhea	8 (47%)	5 (45%)	0 (0%)	3 (100%)
Skin rash	7 (41%)	4 (36%)	2 (67%)	1 (33%)
Paronychia	6 (35%)	3 (27%)	0 (0%)	3 (100%)
Asthenia	4 (24%)	4 (36%)	0 (0%)	0 (0%)
Constipation	3 (18%)	2 (18%)	1 (33%)	0 (0%)
Mucositis	2 (12%)	1 (8%)	0 (0%)	1 (33%)
Dry skin	1 (6%)	1 (9%)	0 (0%)	0 (0%)
Corneal erosion	1 (6%)	1 (9%)	0 (0%)	0 (0%)
Urinary tract infection	1 (6%)	1 (9%)	0 (0%)	0 (0%)
Nausea	1 (6%)	0 (0%)	0 (0%)	1 (33%)
Itch	1 (6%)	0 (0%)	0 (0%)	1 (33%)
Any	13 (76%)	8 (73%)	2 (67%)	3 (100%)
